# New genetic variants associated with major adverse cardiovascular events in patients with acute coronary syndromes and treated with clopidogrel and aspirin

**DOI:** 10.1038/s41397-021-00245-5

**Published:** 2021-06-22

**Authors:** Xiaomin Liu, Hanshi Xu, Huaiqian Xu, Qingshan Geng, Wai-Ho Mak, Fei Ling, Zheng Su, Fang Yang, Tao Zhang, Jiyan Chen, Huanming Yang, Jian Wang, Xiuqing Zhang, Xun Xu, Huijue Jia, Zhiwei Zhang, Xiao Liu, Shilong Zhong

**Affiliations:** 1 BGI-Shenzhen, Shenzhen, 518083 China; 2 China National GeneBank-Shenzhen, BGI-Shenzhen, Shenzhen, 518083 China; 3 School of Biology and Biological Engineering, South China University of Technology, Guangzhou, 510006 China; 4 BGI-tech, BGI-Wuhan, Wuhan, 430075 Hubei China; 5 Guangdong Provincial People’s Hospital, Guangdong Academy of Medical Sciences, Guangzhou, China; 6 Guangdong Provincial Key Laboratory of Coronary Heart Disease Prevention, Guangdong Cardiovascular Institute, Guangdong Provincial People’s Hospital, Guangdong Academy of Medical Sciences, Guangzhou, Guangdong 510080 P.R. China; 7 James D. Watson Institute of Genome Sciences, Hangzhou, 310058 China; 8 Institute of Biopharmaceutical and Health Engineering, Tsinghua Shenzhen International Graduate School, Tsinghua University, Shenzhen, 518055 China; 9 Department of Pharmacy, Guangdong Provincial People’s Hospital, Guangdong Academy of Medical Sciences, Guangzhou, Guangdong 510080 P.R. China

**Keywords:** Genetic association study, Cardiovascular diseases, Outcomes research

## Abstract

Although a few studies have reported the effects of several polymorphisms on major adverse cardiovascular events (MACE) in patients with acute coronary syndromes (ACS) and those undergoing percutaneous coronary intervention (PCI), these genotypes account for only a small fraction of the variation and evidence is insufficient. This study aims to identify new genetic variants associated with MACE end point during the 18-month follow-up period by a two-stage large-scale sequencing data, including high-depth whole exome sequencing of 168 patients in the discovery cohort and high-depth targeted sequencing of 1793 patients in the replication cohort. We discovered eight new genotypes and their genes associated with MACE in patients with ACS, including *MYOM2* (rs17064642), *WDR24* (rs11640115), *NECAB1* (rs74569896), *EFR3A* (rs4736529), *AGAP3* (rs75750968), *ZDHHC3* (rs3749187), *ECHS1* (rs140410716), and *KRTAP10-4* (rs201441480). Notably, the expressions of *MYOM2* and *ECHS1* are downregulated in both animal models and patients with phenotypes related to MACE. Importantly, we developed the first superior classifier for predicting 18-month MACE and achieved high predictive performance (AUC ranged between 0.92 and 0.94 for three machine-learning methods). Our findings shed light on the pathogenesis of cardiovascular outcomes and may help the clinician to make a decision on the therapeutic intervention for ACS patients.

## Introduction

As a standard treatment procedure for patients suffering from ACS and those undergoing PCI with stenting, dual-antiplatelet therapy (DAPT) with clopidogrel in addition to aspirin, significantly reduces the risk of adverse cardiac events in patients [1]. However, the pharmacodynamic response to DAPT varies substantially among patients [2].

Studies have reported the associations of several gene polymorphisms (CYP2C19*2, CYP2C19*3, CYP2C9*2, *PON1* Q192R, and *ABCB1* C3435T) with cardiovascular outcomes in patients with ACS and those undergoing PCI [3–8]. However, there are meta-analysis studies failed to support the associations of *CYP2C19* and cardiovascular events [9, 10]. In summary, these studies about the effects of single-nucleotide variants (SNPs) on clinical outcomes were inconsistent and inconclusive. Meanwhile, most of the previous studies were conducted in western populations, Asians have very different genotype distributions of *CYP2C19* and *PON1* Q192R, for example, the allele frequency of CYP2C19*2 was much higher in the Asian population (35%) than Africans and Caucasians (15%) [11], CYP2C19*3 and CYP2C19*17 in Chinese population was far lower than other populations, and the frequency of the *PON1* 192Q allele in Chinese patients is also relatively lower than in Caucasians [12], in whom relevant studies based on large samples are scarce. Therefore, it is very necessary to identify the association of genetic polymorphisms with cardiovascular events and offer valuable information for intervention in Han Chinese population. Moreover, previous studies on cardiac adverse events after PCI mainly focused on a few specific genotypes or genes [3–10]. Therefore, the majority of the hereditability in affecting cardiovascular events remains unexplained, and other important genetic determinants have yet to be identified. Genome-wide studies, such as whole-exome sequencing, should be conducted to enhance the understanding of this research field.

In the present study, we combined whole-exome and targeted sequencing to investigate the genetic factors associated with the major adverse cardiovascular events (MACE) among patients receiving clopidogrel and aspirin treatments after PCI. The logistic regression and Cox proportional hazard models were used to analyze 1961 samples in two independent cohorts with detailed clinical information, respectively. This process aimed to evaluate the previously reported cardiovascular outcome-related loci and discovered novel genes/alleles associated with the effect of treatment with clopidogrel and aspirin on 18-month MACE in the Han Chinese ethnic group. In addition, we performed the gene-based analysis to further replenish the candidate gene set that maybe missing in single-variant association analysis. To date, machine-learning methods have not been substantially applied to develop effectively predictive prognostic classifiers for adverse cardiovascular events. Thus, we developed SVM classifier to predict the occurrence possibility of 18-month MACE, as well as to provide a target for therapeutic intervention of patients with ACS.

## Materials and methods

### Study population and design

In this study, we included a total of 1961 patients with acute coronary syndromes (ACS) undergoing PCI obtained through Guangdong General Hospital in China from 2009 to 2012. These patients were treated with 12 months of dual-antiplatelet therapy with clopidogrel in addition to aspirin following stent implantation. All patients had detailed baseline and follow-up information during 18-month follow-up periods. Patient information was collected based on inpatient and outpatient hospital visits, and telephone contacts with the patients or their family at 1-, 6-, 12-, and 18-months following discharge. During the 18-month follow-up period, trained staff systematically recorded medical conditions of each patient to determine the occurrence of major adverse cardiovascular events (MACE). An experienced cardiologist adjudicated the end point through review of source documents obtained from medical records. The major adverse cardiovascular events (MACE) as a composite endpoint included cardiovascular death, myocardial infarction (MI), stroke (CT or MR scan confirmed) and repeated revascularization (RR). Repeat revascularization included target vessel revascularization–PCI, nontarget vessel revascularization–PCI, and coronary artery bypass grafting (CABG).

A two-stage analysis was performed in this study. In the discovery cohort, we randomly selected 168 patients for whole-exome sequencing from the total 1021 individuals who have been diagnosed with ACS, have undergone PCI operation in Guangdong General Hospital, and have taken clopidogrel for treatment from 2009 to 2010, of which 51 had MACE end point during the 1-year follow-up period and 117 did not have any clinical events during 18-month follow-up periods. In the replication cohort, we performed targeted sequencing for all 1793 hospitalized patients with ACS and receiving PCI from 2010 to 2012. As replication, 1703 samples went into subsequent analysis after sample quality control, of which 123 had MACE end point during the 18-month follow-up period and 1580 did not have any clinical events during that period. This study has been registered at http://www.chictr.org.cn on 8 March 2011 (registration number: ChiCTR-OCH-11001198).

This study was approved by both the Guangdong general hospital ethics committee and the BGI ethics committee. All protocols were conducted in compliance with the Declaration of Helsinki. All participants provided written informed consent to take part in the study.

### Sample sequencing in two stages

We sequenced the whole exome of 168 patients in the discovery cohort. Genomic DNA for each sample was used to produce each exome-captured library with the NimbleGen SeqCap EZ Exome (44MB, Roche) array. Then each captured library was independently sequenced on the Illumina Hiseq 2000 platform. Each sample was designed to get high-quality bases with coverage of >90×.

We sequenced the 6-MB targeted region of 1793 patients in the replication cohort. The targeted region consisted of three parts. The first part was the top associated SNVs with *P* < 0.05 in the discovery stage. The second part was top genes with *P* < 0.05 in gene-based test in the discovery study. Gene-based analysis was performed using the Fast Association Tests (FAST) tool [13], which includes a series of gene-based methods. Four algorithms, including (i) Gene-Wide Significance test (GWiS); (ii) MinSNP-p and MinSNP-gene; (iii) Versatile Gene-Based Test for Genome-wide Association (VEGAS); (v) the Gates test (GATES), were used to test the gene-based association with MACE. The significant gene (*P* < 0.05) in at least three of the four tests was selected for the targeted region design. The third part was 49 reported genes within the pharmacokinetic and pharmadynamics pathway of clopidogrel, aspirin, statin, or beta-blockers. All the three parts were combined and merged into 6MB target regions. Then the targeted region for each sample was sequenced on Complete Genomics (CG) platform [14] with high quality and coverage.

### Sample quality control

We required the samples to meet these criteria: (i) average sequencing depth ≥90× in exome-sequencing stage, (ii) average sequencing depth ≥30× in targeted sequencing stage, (iii) genotype calling rate ≥90%, (iv) not-existing population stratification by performing principal component analysis (PCA) analysis that was performed via the multidimensional scaling (MDS) procedure implemented in PLINK v 1.07 [15], and (v) not be duplicates or first-degree relatives while evaluating pairwise by identity by descent (IBD). After quality control filtering, 90 samples from the targeted sequencing stage were excluded from subsequent analysis.

### Alignment, variant calling, and quality control

For Illumina exome-sequencing data, reads were mapped to human genome reference assembly (hg19, GRCh37) with SOAP2 [16] and variants were detected by SOAPsnp [17]. The high-quality Illumina SNVs that we defined for each individual had to meet the following condition: sequenced quality ≥Q20, sequencing depth ≥8× and ≤500×, and depth of nonreference allele ≥4×.

For CG-targeted sequencing data, sequence reads were also aligned to human reference genome hg19 and variations were detected using the CG analysis toolkit (CGATools) which is available at CG website (http://cgatools.sourceforge.net/).

After all initial SNV calls from Illumina and CG platforms were generated, further filtering was performed to identify high-confidence SNVs. We required SNVs from discovery and replication studies to meet two conditions: (i) genotype calling rate ≥90%, (ii) minor allele frequency (MAF) ≥ 0.01, and (iii) Hardy–Weinberg equilibrium (HWE) *P* > 1.0 × 10^−6^.

To evaluate the data quality, we compared the genotypes from the sequencing data with the genotypes called from the genotyping arrays. In the discovery study, the average genotype concordance is 98.6% by comparing 5 genotypes overlapping in the exome-sequencing data and genotyping array data in 126 samples (Table S1). In the replication study, the whole-genome sequencing result of the YanHuang (YH) sample was obtained using CG platform. We evaluated the CG data quality by comparing the whole-genome sequencing result and genotyping results in the YH sample and the genotype concordance was 99.5%.

### Statistical analysis

Analysis was performed using PLINK (version 1.07) [15] and R (version 3.2.3, http://www.R-project.org/). The demographic and clinical characteristics were summarized using counts (percentages) for the categorical variables (e.g., sex, previous MI, and diabetes mellitus) and mean (standard deviation, SD) for the continuous variables (e.g., age, BMI). A multivariate Cox proportional hazard model [18] was used to calculate the significance by comparing the baseline demographic and clinical characteristics between the groups with and without MACE in the follow-up periods of 18-months. Baseline variables and genetic variants explanation for MACE incidence during the 18-month follow-up were calculated by using a regression-based approach as implemented in the SOLAR-Eclipse version 8.1.1 software (http://solar-eclipse-genetics.org/index.html).

In the discovery cohort, we applied the logistic regression to calculate the P values and odds ratio (OR) of SNPs on the clinical end points by adjusting for 17 variables, including the first four principal components PC1–PC4, three demographic (sex, age, and BMI) and 10 clinical variables (e.g., diabetes mellitus, hypertension, and previous MI). In the replication cohort, we use the multivariate Cox proportional hazard regression to model the survival time and the incidence of MACE by adding SNPs and the same 17 adjustment variables as covariates to evaluate the hazard ratio (HR) and *P* value for each SNP.

The Cox model is expressed by the hazard function denoted by h(t). Briefly, the hazard function can be interpreted as the risk of MACE at time t. It can be estimated as follows:

*h*(*t*)=*h*_0_(*t*)×exp(*b*_1_*x*_1_ + *b*_2_*x*_2_ + …+*b*_*p*_*x*_*p*_)

where

- *t* represents the survival time of MACE, ranging from 0 to 18 months.
- *h*(*t*) is the hazard function determined by a set of p covariates (*x*_1_, *x*_2_,…, *x*_p_), p covariates were SNPs and the 17 adjustment variables in this study.
- the coefficients (*b*_1_, *b*_2_,…, *b*_p_) measure the impact (i.e., the effect size) of covariates.
- the term *h*_0_ is called the baseline hazard. It corresponds to the value of the hazard if all the *x*_*i*_ are equal to zero (the quantity exp(0) equals 1). The ‘t’ in h(t) reminds us that the hazard may vary over time.

We fit the multivariate Cox proportional hazards regression model with coxph() function in survival package in R. The coxph function was written as

>coxph(Surv(time, status) ~age + sex + … + genotypes, data = MACE)

Finally, we performed a meta-analysis of these two datasets, via inverse-variance weighted fixed effect meta-analysis method [19] based on log hazard ratio and standard error. Before that, the Cox regression model was also implemented for the discovery cohort to get the HR for meta-analysis.

Power analysis was used to investigate if we have enough power to detect the associated SNPs in the sample size of the replication cohort. R package survSNP (version 0.23.2) was used to evaluate the power for SNPs with different HR or MAF.

We estimated a variant as ‘deleterious’ (or ‘functional’ or ‘damaging’) or ‘benign’ (or ‘nonfunctional’ or ‘neutral’) using Polyphen2 [20] and combined annotation-dependent depletion (CADD) [21] methods. A variant with CADD score >15 was defined as “damaging” as recommended by Itan et al. [22].

### Gene expression analysis

We mined four publicly available genome-wide expression data sets from Gene Expression Omnibus (GEO) database in NCBI, including GSE27962 [23], GSE48060 [24], GSE7487 [25] and GSE47495 [26]. These datasets recorded gene expression data of cardiac remodeling after myocardial infarction or myocardial infarction-induced heart failure. After downloading the four publicly available datasets, we first detected the expression data distribution for the eight genes in this study, then we applied log transformation and quantile normalization to the expression data if they were not normally distributed. To compare the gene expression difference between case groups (mainly myocardial infarction (MI) operation) and control groups (sham operation), we used unpaired Student’s *t*-test to compute the significance as GEO website recommended. Data are presented as mean ± SE (standard error). Finally, we corrected for multiple testing of the eight genes and genes having a threshold of *P* < 0.00625 = 0.05/8 were considered to be differentially expressed between two groups.

### *ECHS1* plasma experiments

We randomly selected ACS patients with heart failure (HF) symptoms at NYHA stage II or less (*n* = 61), stage III or IV (*n* = 89) from an independent study cohort. *ECHS1* protein levels were measured in plasma sample using sandwich enzyme-linked immunosorbent assays (ELISA) (ECHS1 ELISA kit, Action-award Biotech co. Ltd., Guangzhou, China) and a Multiskan GO Microplate Reader (Thermo Scientific Inc., USA). The comparison of parameters and *ECHS1* protein levels between patients with HF at stage II or less (*n* = 61) and patients at stage III or IV (*n* = 89) was performed using the logistic regression analysis. Because the distribution of *ECHS1* protein levels was skewed, logarithmic transformation was performed before analysis.

### Prediction of 18-month MACE

This study used three machine-learning models, including support vector machine (SVM) method [27], Light Gradient Boosting Machine (LightGBM) [28] and XGBoost [29], to predict the binary phenotype—whether MACE occurred in a patient during the 18-month follow-up. The prediction analysis was completely independent from the association analyses, and all the genetic and nongenetic factors were input for analysis. There are 14,253 SNPs overlapped between the discovery and replication cohorts. After LD pruning (PLINK -indep-pairwise 50 10 0.2), 7246 independent SNPs remained. These 7246 independent SNPs as well as 20 clinical factors entered into the three machine-learning models for prediction. The whole dataset was split into 80% and 20% subsets for model training and testing, using stratified folds by balancing the percentage of samples for each class in train and test. We applied a fivefold cross-validation procedure on the train dataset to select the best hyperparameters. One fold was used as the validation set and the other four folds were involved in parameter tuning and model constructing. We repeated the process until each one of the five folds was used as the validation set once. Then we constructed the final model on the train data using the best parameters and calculated the feature importance. In each model, the factors with feature importance over zero were selected for prediction. Finally, we estimated the predictive performance based on the final model on the test dataset. Feature importance was also output to help us pinpoint the important factors that contributed to 18-month MACE. Three models were utilized so that we were able to compare their prediction performances, which could potentially reduce the bias from simply assessing one model. All the modeling procedures were conducted in Python (v3.7.8) with scikit-learn package and publicly available packages.

## Results

### Characteristics of the patients

Two independent cohorts were recruited and comprised 1961 patients with ACS who underwent PCI and treated with clopidogrel and aspirin for 6–12 months in accordance with the consensus guidelines. After performing sample quality control, 168 patients were included in the discovery study and 1703 patients remained in the replication study (Table S2 and S3). For the total 1871 patients, the average age was 63.3 (±10.7) years, 357 (19.1%) were women, 174 (9.3%) had a MACE endpoint during the 18-month follow-up period. Table 1 shows the association of demographic and clinical characteristics with MACE. MACE was associated with increasing age (65.84 ± 10.17 vs. 63.07 ± 10.74, *P* < 0.001), hypertension (68% vs. 56%, *P* < 0.001), and high creatinine (100.9 ± 85.96 vs. 87.03 ± 44.84, *P* < 0.001) (Table 1). These combined clinical variables explained 5.80% of occurrence of the MACE, thus suggesting potentially substantial genetic contribution.

**Table 1:** Demographic and clinical characteristics contributed to MACE in ACS patients (*n* = 1871).

Characteristics	β (95% CI)	*P value*	Variants explanation
Age – yrs, mean (±s.d.)	1.025(1.010–1.040)	<0.001***	0.94%
Sex, Men – no. (%)	1.073(0.7404–1.554)	0.71	
BMI –kg/m^2^, mean (±s.d.)	1.016(0.9593–1.077)	0.58	
**Risk factors, n (%)**			
Previous MI	1.075(0.7905–1.461)	0.65	
Diabetes mellitus	1.322(0.9657–1.809)	0.08	
Hypertension	1.689(1.227–2.325)	<0.001***	0.87%
**Medications used before event**			
ACEI_ARB	1.356(0.8811–2.086)	0.17	
BBI	1.178(0.7313–1.896)	0.5	
CCB	1.231(0.8747–1.733)	0.23	
PPI	1.269(0.9425–1.709)	0.12	
Statins	1.069(0.3967–2.882)	0.89	
**Blood metabolites level**			
HDLC, mmol/L	0.8664(0.4562–1.646)	0.66	
LDLC, mmol/L	0.8714(0.7305–1.040)	0.13	
Triglycerides, mmol/L	0.9466(0.8074–1.110)	0.5	
HbA1c, % total hemoglobin	0.8917(0.7732–1.028)	0.12	
ALT, U/L	1.002(1–1.005)	0.06	
AST, U/L	0.9973(0.993–1.002)	0.21	
CREA, umol/L	1.003(1.001–1.005)	<0.001***	0.73%
CK, U/L	0.9994(0.9987–1)	0.28	
CKMB, U/L	0.9813(0.9622–1.001)	0.68	

*P* value was calculated by multivariate Cox regression analysis. *P* < 0.05 was considered a statistically significant difference. β represents exp(coefficient).

BMI, denotes Body-mass index; MI, myocardial infarction; ACEI, angiotensin-converting-enzyme inhibitor; ARB, angiotensin receptor blocker; BBI, β-blockers inhibitors; CCB, calcium channel blockers; PPI, proton pump inhibitors; HDLC, highdensity lipoprotein cholesterol; LDLC, low-density lipoprotein cholesterol; HbA1c, hemoglobin A1c; ALT, alanine aminotransferase; AST, aspartate aminotransferase; CREA, creatinine; CK, creatine kinase and CKMB, creatine kinase MB.

Signif. codes: 0 ‘***‘ 0.001 ‘**‘ 0.01 ‘*‘ 0.05 ‘.’ 0.1 ‘ ‘ 1.

### Two-stage association study

**Fig. 1:**
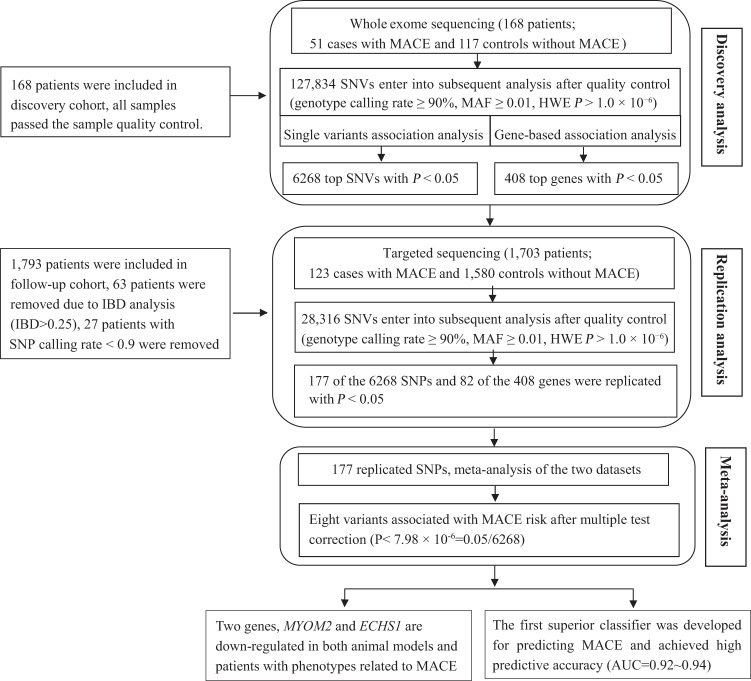
Description of the study design. First, we performed whole exome sequencing of 168 patients and 51 out of them had MACE end point. After quality filtering, a total of 127,834 variants were subjected to single variant association analysis and 6268 variants showed nominal association (*P* < 0.05). Gene-based association analyses identified 408 genes associated with MACE (*P* < 0.05). As validation, the 6MB targeted region including 6268 top SNPs and 408 top genes was further analyzed in additional 1703 patients through multivariable Cox regression analysis. A total of 177 SNPs and 82 genes were replicated in validation datasets. Finally, we performed meta-analysis of the two-stage associations and identified eight genetic variants contributed to MACE (*P* < 7.98 × 10^−6^ = 0.05/6268). Then, we performed functional analysis on the eight significant SNPs or genes; further, we developed the first superior classifier for predicting MACE.

The study design and total workflow is shown in Fig. 1. In the discovery study, we deep-sequenced the whole exome of the 168 ACS patients with a mean coverage of approximately 210× (Table S4, Fig. S1). All cases with MACE and controls without MACE were ethnically and genetically well matched (Fig. S2). A total of 127,834 SNPs passed the quality control for the single-variant association analysis. Logistic regression analysis determined 6268 SNPs associated with MACE with *P* < 0.05 adjusting for the covariates **(**Fig. S3). Gene-based association analysis identified 408 genes associated with MACE at the *P* < 0.05 level (Table S5).

To further replicate the identified associations in the discovery cohort and increase the statistical power of the present study, we performed a replication analysis in an independent cohort of 1703 patients using targeted sequencing. In the replication analysis, a total of 6-MB (million bases) targeted regions consisting of SNP/gene associations discovered above and previously reported, were sequenced with approximately 94× coverage for each individual (Table S5, Fig. S1). After performing quality control on the samples and variants, 28,316 SNPs entered the subsequent analyses (Fig. S3**)**. Then, we performed replication analysis on the 6268 SNPs and 408 genes and found 177 replicated SNPs for MACE with *P* < 0.05 both in the discovery and replication studies. Although no SNPs reached the multiple test-adjusted significance in the replication cohort, there are eight SNPs left after multiple-test correction (*P* < 7.98 × 10^−6^ = 0.05/6,268; Table 2) while performing the meta-analysis of two-stage data.

**Table 2:** Identifying eight genetic variants contributed to MACE.

Chr.	Gene	Variant ID	Stage^a^	Alleles	RAF	OR/HR^+^ (95% CI)	*Pvalue*	Meta *Pvalue*
8	*MYOM2*	rs17064642	i	C/T	0.083	6.48(1.83–15.01)	2.95 × 10^−4^	1.84 × 10^−7^
			ii	C/T	0.064	2.40(1.59–3.63)	3.26 × 10^−5^	
16	*WDR24*	rs11640115	i	G/A	0.712	3.13(1.25–3.85)	2.13 × 10^−3^	3.21 × 10^−7^
			ii	G/A	0.694	2.08(1.47–2.88)	2.56 × 10^−5^	
8	*NECAB1*	rs74569896	i	G/A	0.138	2.87(1.51–5.45)	3.87 × 10^−3^	1.31 × 10^−6^
			ii	G/A	0.154	1.90(1.39–2.61)	5.91 × 10^−5^	
8	*EFR3A*	rs4736529	i	G/C	0.060	3.16(1.04–9.61)	4.25 × 10^−2^	2.52 × 10^−6^
			ii	G/C	0.060	2.41(1.45–4.27)	1.95 × 10^−5^	
7	*AGAP3*	rs75750968	i	A/T	0.036	3.38(1.05–10.9)	2.90 × 10^−2^	4.85 × 10^−6^
			ii	A/T	0.021	3.21(1.83–5.66)	5.21 × 10^−5^	
3	*ZDHHC3*	rs3749187	i	A/G	0.030	6.12(1.17–34.01)	4.12 × 10^−2^	5.19 × 10^−6^
			ii	A/G	0.027	2.99(1.26–4.14)	3.37 × 10^−5^	
10	*ECHS1*	rs140410716	i	T/C	0.018	12.01(1.10–104.1)	1.49 × 10^−2^	6.88 × 10^−6^
			ii	T/C	0.015	3.32(1.84–5.99)	6.54 × 10^−5^	
21	*KRTAP10–4*	rs201441480	i	A/C	0.024	7.25(1.44–36.56)	3.12 × 10^−3^	7.26 × 10^−6^
			ii	A/C	0.011	3.86(1.87–7.99)	2.72 × 10^−4^	

Chr., chromosome; RAF, risk allele frequency; OR, odds ratio; HR, hazard ratio; CI, confidence interval. Alleles are shown as risk allele/reference allele.

^a^Stage i: exome sequencing for 168 individuals; Stage ii: targeted sequencing for 1703 individuals. ^**+**^OR in Stage i and HR in Stage ii.

### Identify single variants and genes associated with 18-month MACE

**Fig. 2:**
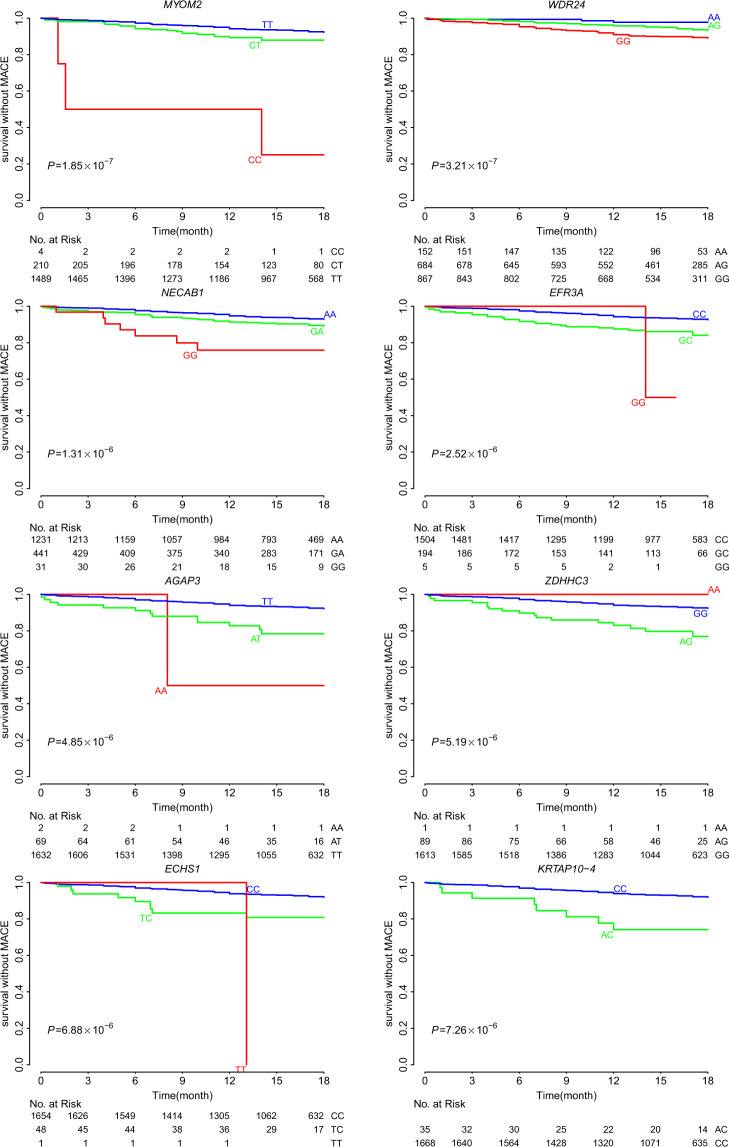
Event-free survival over 18 months of follow-up in 1703 patients with ACS. Cumulative probabilities of survival without MACE according to gene polymorphisms: *MYOM2* (rs17064642), *WDR24* (rs11640115), *NECAB1* (rs74569896), *EFR3A* (rs4736529), *AGAP3* (rs75750968), *ZDHHC3* (rs3749187), *ECHS1* (rs140410716), and *KRTAP10-4* (rs201441480). The red, green, and blue colors represented the genotypes containing two, one or zero copy of the risk allele, respectively.

The most significant SNP associated with 18-month MACE was rs17064642 at *MYOM2* (*P* = 1.84 × 10^−7^; Table 2). Carriers of the CC/CT genotype had higher MACE occurrence rates during the 18 months of follow-up compared with noncarriers (17.8% vs. 8.1%; HR, 2.76; 95% CI, 1.98–3.87) (Fig. 2). Rs17064642 has moderate linkage disequilibrium (LD) of r^2^ = 0.57 (East Asians) with the missense SNP rs34823600 and these two SNPs are located only 16 bp apart (Table S6). Similarly, rs34823600 was also significantly contributed to MACE (HR 2.25, *P* = 5.32 × 10^−4^). Rs17064642 and rs34823600 both coincide with enhancer markers (H3K4me1_Enh and H3K27ac_Enh) in four tissues, especially in heart cell types (right atrium, left ventricle and right ventricle), suggesting this locus may function as an enhancer in heart tissue (Fig. S4).

The other seven significant associations included *WDR24* (rs11640115), *NECAB1* (rs74569896), *EFR3A* (rs4736529), *AGAP3* (rs75750968), *ZDHHC3* (rs3749187), *ECHS1* (rs140410716), and *KRTAP10-4* (rs201441480) (Table 2). At *WDR24*, we identified two SNPs associated with MACE, rs11640115 (HR 2.08, *P* = 3.21 × 10^−7^) and rs763053 (HR 1.92, *P* = 8.02 × 10^−7^), which were in high LD (D′ = 0.98, r² = 0.96, Table S6). According to public GTEx [30] databases, both rs11640115 and rs763053 at *WDR24* showed significant eQTL associations with gene *WDR90* (*P* = 1.16 × 10^−6^) in the heart tissues, including heart left ventricle and heart atrial appendage (Table S6**)**. Evidence from GSE48060 [24] dataset confirmed that *WDR90* transcription was associated with long-term recurrent events following first-time MI (0.122 ± 0.031, *P* = 4.78 × 10^−3^, Table S7 and S8). Similarly, we also identified two SNPs at *NECAB1* associated with MACE, rs74569896 (HR 1.90, *P* = 1.31 × 10^−6^) and rs73694346 (HR 1.72, *P* = 4.86 × 10^−5^), which were in strong LD (D′ = 0.96, r² = 0.92). Rs4736529 at *EFR3A* increased the risk of occurring MACE and expression of gene *EFR3A* was significantly upregulated in the coronary artery disease (CAD) patients compared with the control group [31]. However, *EFR3A* showed significantly decreased expression in the left ventricular remodeling in swine after myocardial infarction cardiac by analyzing the GEO database GSE27962 [23] (*P* = 6.94 × 10^−4^). SNP rs75750968 locates in gene *AGAP3*, which relates to GTP binding and GTPase activator activity. Rs74569896 located in *NECAB1* binds to the protein *GATA3* in ChIP-Seq experiments (ENCODE Project Consortium, 2011) and lack of *GATA3* results in conotruncal heart anomalies in mouse [32]. Rs201441480 at *KRTAP10-4*, which is a missense alteration that was predicted to be damaging by both Polyphen2 [20] and CADD [21] methods, significantly increased the risk of MACE (HR 3.86, *P* = 7.26 × 10^−6^). Together, we discovered eight novel genetic variants associated with MACE and achieved an average of 94% GWAS statistical power for the eight SNPs, of which, 99.7% statistical power for rs11640115 at *WDR24* (Table S9, Fig. S5). Fig. 2 showed cumulative proportions of individuals without MACE over 18 months of follow-up under the eight polymorphisms.

Since we got the whole-exome and targeted region sequencing data, we further performed the gene-based analysis and confirmed the significance of the eight genes (Table 3). Together, we identified eight novel genetic variants and their genes contributed to 18-month MACE.

**Table 3:** Gene-based results showing eight genes contributed to MACE.

Gene	Stage i		Stage ii	
	Gene. *Pvalue*	TopSNP. *Pvalue*	Gene. *Pvalue*	TopSNP. *Pvalue*
*MYOM2*	3.54E-02	2.95E-04	2.75E-02	8.87E-05
*WDR24*	7.33E-03	2.13E-03	2.64E-04	5.36E-05
*NECAB1*	8.82E-02	2.77E-03	6.73E-03	1.32E-04
*EFR3A*	4.20E-02	2.32E-02	7.43E-03	3.41E-05
*AGAP3*	4.16E-02	1.93E-02	2.17E-02	1.26E-03
*ZDHHC3*	4.12E-02	1.25E-02	3.11E-04	3.21E-05
*ECHS1*	1.30E-02	1.49E-02	1.70E-04	8.73E-05
*KRTAP10-4*	3.23E-02	3.12E-03	8.18E-03	7.94E-04

Stage i, exome sequencing for 168 individuals; Stage ii, targeted sequencing for 1703 individuals.

### Biological implications of variants associated with 18-month MACE

To further explore the functional evidence of the eight genes that contribute to 18-month MACE, we mined several publicly available genome-wide expression data sets from the GEO database (Table S8) [23–26], which recorded cardiac remodeling data after myocardial infarction (MI). We found that two of eight genes, *MYOM2* and *ECHS1*, showed abundant evidences of decreased expression in cases with major adverse cardiac events, either in previous literatures or in our data analysis. M-protein (myomesin-2) encoded by *MYOM2* or total myomesin is downregulated in cardiac hypertrophy in rats [33], in acute myocardial infarction (AMI) patients [34], or in chronic heart failure [35]. Similarly, *ECHS1*, as an ischemic post-conditioning (PostC) modified protein, showed significantly decreased expression in the cardiac remodeling group after MI compared with the sham group, by analyzing the two different groups’ expression data from GEO database GSE7487 [25] (7.748 ± 4.319 vs. 4.214 ± 1.668, *P* = 5.46 × 10^−6^) and GSE47495 [26] (11.89 ± 0.11 vs. 12.13 ± 0.04, *P* = 7.28 × 10^−4^, Table S6 and S7). Meanwhile, we compared the plasma *ECHS1* protein levels in ACS patients with HF symptoms at New York Heart Association (NYHA) stage III or IV (*n* = 89) to those with HF symptoms at NYHA stage II or less (*n* = 61). We found that advanced HF patients showed significantly decreased levels of *ECHS1* protein (215.18 ± 115.67 for stage II or less vs. 161.84 ± 76.67 for stage III or stage IV, *P* = 0.0012) (Table S10, Fig. S6).

### Predictive effectiveness of 18-month MACE

**Fig. 3:**
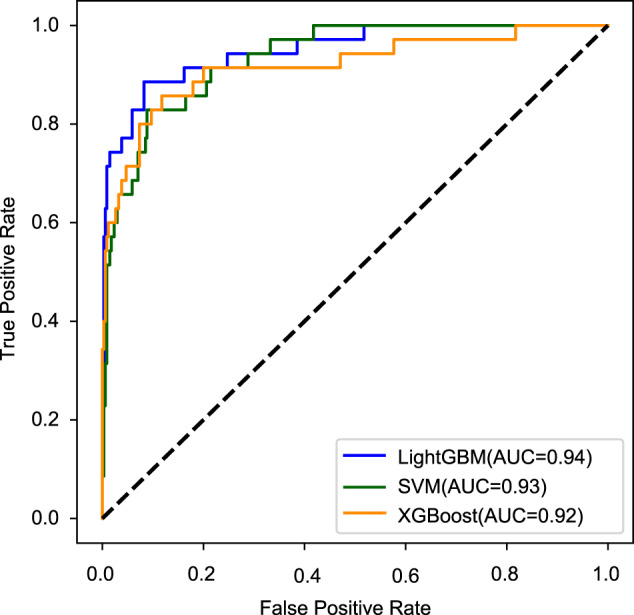
Receiver-operating characteristic (ROC) curves for prediction of 18-month MACE. The predictive effectiveness was evaluated by three machine learning models, including support vector machine (SVM, green), Light Gradient Boosting Machine (LightGBM, blue) and XGBoost (orange). A total of 7246 independent factors (20 clinical factors and 7226 LD-pruning SNPs) entered into each model for modeling, fitting and prediction.

One of the ultimate objectives for identifying factors associated with MACE is to predict whether MACE occurred during the follow-up period of 18 months for ACS patients receiving PCI. Based on all the genetic and nongenetic factors, including all 20 clinical factors and 14,253 genetic variants that overlapped in two-stage datasets (remaining 7226 SNPs after LD pruning), we constructed predictive models for 18-months MACE using three machine learning methods, including support vector machine (SVM) [27], Light Gradient Boosting Machine (LightGBM) [28], and XGBoost [29]. These three predictive models showed similar performances, with AUCs ranging between 0.92 and 0.94 from the average of fivefold cross-validation (Fig. 3). We generated the feature importance score plot from the best-performing model LightGBM and the result was listed in Table S11. Among the 7246 independent factors (20 clinical factors and 7226 LD-pruning SNPs), 195 factors were selected as predictors of 18-month MACE with importance score over zero in LightGBM model. Three clinical factors, creatinine (importance score 776), age (score 676) and hypertension (score 46), were identified as important predictors (Table S11), which is consistent with our observational correlation findings (Table 1). In addition, seven of the eight significant genes were also selected as predictors (Table S11). We further investigated the significance of the SNPs selected by LightGBM for the prediction in our genetic association study. We found that those SNPs that effectively contributed to the prediction exhibited significantly lower *p*-value enrichment (Fig. S7). Thus, the present study developed the first superior classifier for predicting 18-month MACE by selecting the most informative factors that independently contributed to MACE.

## Discussion

To our knowledge, the present study is the first genome-wide and large-scale association analysis that integrates whole-exome and targeted sequencing to identify novel genetic variants associated with MACE in patients with clopidogrel and aspirin treatment after PCI. We also investigated the associations of five commonly reported SNPs [4, 6–8, 36–38] with MACE. Our meta-analysis of the two-stage sequencing data confirmed that *PON1* (*P* = 0.0036) but not *CYP2C19* genetic variants contributed to cardiovascular outcomes in Han Chinese patients (Table S12). Moreover, we identified eight novel genetic variants associated with MACE: *MYOM2* (rs17064642), *WDR24* (rs11640115), *NECAB1* (rs74569896), *EFR3A* (rs4736529), *AGAP3* (rs75750968), *ZDHHC3* (rs3749187), *ECHS1* (rs140410716), and *KRTAP10-4* (rs201441480).

To further explore the functional evidence of the eight genes that contribute to 18-month MACE, we searched all literatures referring to the eight genes and cardiovascular diseases in PubMed database, as well as mined previously reported publicly available GEO databases. Among the eight genes contributed to MACE, we find the expression levels of *MYOM2* and *ECHS1* are evidently downregulated in cases subjected to adverse cardiac events compared with normal controls. Interestingly, many clues reveal that *MYOM2* and *ECHS1* play an important role in heart function. First, *MYOM2* encodes the M-protein, which is also known as Myomesin-2. Myomesin-2 is the primary myosin M-band cross-linking protein and binds titin in a complex with obscurin/obs1. The protein is key to normal heart function, as evidenced by the associations between heart failure and low expression. Myomesin-2 showed decreased expression in multiple heart diseases or heart attack. An animal model of cardiac hypertrophy driven by the thyroid hormone (T3) in rats showed that the downregulated *MYOM2* causes significant contractile dysfunction (*P* < 0.05) [33]. In addition, it has been reported that myomesin (encoded by both *MYOM1* and *MYOM*2) protein levels decrease in acute ischemia and in chronic heart failure [35]. A proteomic analysis agrees with previous reports that the level of myomesin-2 in cardiac tissue is decreased in AMI patients (*n* = 10) compared with control cases (*n* = 11) [34]. Second, the *ECHS1* protein (short-chain enoyl-CoA hydratase) is a multifunctional mitochondrial enzyme with several functions in β-oxidation of short- and medium-chain fatty acids, as well as in isoleukine and valine metabolism. A previous study reported that the mitochondrial protein *ECHS1* could regulate cellular ATP consumption/production and influence the defense response to myocardial ischemic stress [39]. Haack et al. reported that *ECHS1* deficiency causes mitochondrial encephalopathy with cardiac involvement [40]. Third, *ECHS1* showed significantly decreased expression in the cardiac remodeling group after MI compared with the sham group in two GEO datasets GSE7487 and GSE47495. We further confirmed that *ECHS1* contributed to an ischemic heart failure in ACS patients by gene expression experiment. Combining all these evidences, we concluded that *MYOM2* and *ECHS1* deficiency causes dysfunction of cardiac function, which will help us understand the occurrence mechanism of MACE.

Apart from identifying the novel genotypes and genes associated with MACE, we improved the prediction of MACE by developing a novel classifier for 18-month MACE, when compared with previous studies [41–44]. Previous studies were mainly based on clinical features; no comprehensive and complete genetic makers were available for the prognostic classification of MACE. In the current study, we included all candidate genetic variants of MACE to well construct predictive models. We confirmed genetic variants that significantly associated with MACE that efficiently contributed to prediction of MACE than other less-associated factors (Fig. S7). In summary, we developed the first fine classifier that combined the clinical factors and multiple independently informative genotypes to predict 18-month MACE and achieved high accuracy (AUCs ranging between 0.92 and 0.94 from three machine-learning methods).

There are two issues that need to be addressed in this study. The first one is that the design of the discovery and the replication studies was different, the discovery stage is designed as the case–control study and the replication stage is designed as the cohort study. There was an obvious time interval between the patient cases and the controls in the discovery study, one is that the event occurred within 1-year old, the other is that the event did not occur within 1.5-years old, which can be clearly divided into case and control, and it is suitable for logical regression analysis. However, the cases and controls were defined as whether the event occurred within 1.5 years or not in the replication study; the multivariate Cox proportional hazards regression could model the survival time and the incidence of MACE simultaneously, thus, Cox proportional model is most suitable for replication study. In summary, this study is a complete and reasonable design that the discovery cohort is a screening for candidate target genes and we designed the 6-MB targeted region for further validation in the replication cohort. Although we used logistic regression analysis for the discovery cohort and performed multivariate Cox analysis for the replication cohort, some associations were still verified with each other and proved the reliability and consistency of our findings. The second issue was about the prognostic effectiveness of MACE classifier, we maybe overestimating the performance of the classifier because we have not got more new available independent samples as test dataset. Further validation and more work still need to be done in larger samples or more populations.

In conclusion, we provide here the first genome-wide, large-scale association analysis on ACS patients receiving clopidogrel and aspirin treatment after PCI. We successfully identified eight novel genes for MACE and found that these genetic variants may regulate the function of nearby genes. Especially, the expressions of *MYOM2* and *ECHS1* are downregulated in both animal models and patients with phenotypes related to MACE. Importantly, we developed the first superior classifier to predict 18-month MACE and achieved high accuracy. These findings will provide clinicians with potential biomarkers for an improved prediction of 18-month MACE and provide new insight on the therapeutics of ACS.

## Supplementary information

Supplementary tables and figures

Table S11. feature importance.xlsx

## Data Availability

All materials and datasets generated and/or analyzed during the current study are available from the corresponding author on reasonable request.
