# Supplementary material for: New genetic variants associated with major adverse cardiovascular events in patients with acute coronary syndromes and treated with clopidogrel and aspirin
Source: Pharmacogenomics J. 2021 Jun 22;21(6):664–72. doi: 10.1038/s41397-021-00245-5 (PMC8602039; doi:10.1038/s41397-021-00245-5)
Supplement: Supplementary file 1 — Supplementary tables and figures [file 41397_2021_245_MOESM1_ESM.docx]

**Supplementary Tables and Figures**

[Table S1. The SNP concordance rate between whole exome sequencing and genotyping chip. 2](#_Toc71729556)

[Table S2. Baseline and clinical characteristics contributed to MACE in discovery cohort (n=168). 3](#_Toc71729557)

[Table S3. Baseline and clinical characteristics contributed to MACE in replication cohort (n=1,703). 4](#_Toc71729558)

[Table S4. Data production summary. 5](#_Toc71729559)

[Table S5. A total of 6M targeted region in replication cohort. 6](#_Toc71729560)

[Table S6. Functional annotation of the eight significant genetic variants. 7](#_Toc71729561)

[Table S7. Eight genes and their differently expression between MI groups and sham groups among four studies. 8](#_Toc71729562)

[Table S8. Detailed information for four GEO datasets. 9](#_Toc71729563)

[Table S9. Statistic power for the eight top SNPs associated with MACE. 10](#_Toc71729564)

[Table S10. Baseline characteristics of ACS patients with heart failure (HF) symptoms at NYHA stage II or less, stage III or IV. 11](#_Toc71729565)

Table S11. 195 features selected with importance score over zero by LightGBM for prediction of 18-months MACE (see attached excel)

[Table S12. Association results of five previously reported SNVs associated with clopidogrel response. 12](#_Toc71729566)

[Figure S1. Depth distribution of sequencing. 13](#_Toc71729567)

[Figure S2. PCA analysis in discovery and replication cohort, respectively. 14](#_Toc71729568)

[Figure S3. QQ plot and Manhattan plot of associations for MACE. 15](#_Toc71729569)

[Figure S4. HaploReg results for the eight significant SNPs associated with MACE. 16](#_Toc71729570)

[Figure S5. Statistics power. 17](#_Toc71729571)

[Figure S6. Compared plasma ECHS1 level in ACS patients with severe HF symptoms (stage III or IV) to those with less serious HF symptoms (stage II or less). 18](#_Toc71729572)

[Figure S7. The QQ plots for the discovery SNPs, replication SNPs, the meta-analysis SNPs and the SNPs selected by LightGBM for prediction of 18-months MACE in this study.. 19](#_Toc71729573)

# Table S1. The SNP concordance rate between whole exome sequencing and genotyping chip.

| \| SNPs \| Samples \| All alleles \| Identical alleles \| Identical rates \| \| --- \| --- \| --- \| --- \| --- \| \| rs662 (*PON1*) \| 124 \| 248 \| 244 \| 98.39% \| \| rs4244285(*CYP2C19*2*) \| 126 \| 252 \| 249 \| 98.81% \| \| rs1128503(*ABCB1*) \| 116 \| 232 \| 227 \| 97.84% \| \| rs1042713(*ADRB2*) \| 114 \| 228 \| 224 \| 98.25% \| \| rs2306283(*SLCO1B1*) \| 123 \| 246 \| 245 \| 99.59% \| \| Average \|  \|  \|  \| 98.58% \| |
| --- | --- | --- | --- | --- | --- | --- | --- | --- | --- | --- | --- | --- | --- | --- | --- | --- | --- | --- | --- | --- | --- | --- | --- | --- | --- | --- | --- | --- | --- | --- | --- | --- | --- | --- | --- |

# Table S2. Baseline and clinical characteristics contributed to MACE in discovery cohort (n=168).

| **Characteristics** | **β (95% CI)** | **Pvalue** | **Variants**  **explanation** |  |
| --- | --- | --- | --- | --- |
| Age – yrs, mean (s.d.) | 1.016(0.9868-1.045) | 0.29 |  |  |
| Sex, Men – no. (%) | 1.846(0.9235-3.689) | 0.08 |  |  |
| BMI –kg m–2, mean (s.d.) | 1.004(0.8983-1.123) | 0.94 |  |  |
| **Risk factors, n (%)** | | | | |
| Previous MI | 0.5928(0.3349-1.049) | 0.07 |  |  |
| Diabetes mellitus | 1.778(1.017-3.112) | 0.04* | 0.2% |  |
| Hypertension | 2.772(1.420-5.412) | 0.003** | 0.8% |  |
| **Medications used before event** | | | | |
| ACEI_ARB | 1.711(0.7298-4.013) | 0.22 |  |  |
| BB | 1.174(0.4663-2.955) | 0.73 |  |  |
| CCB | 1.342(0.7252-2.485) | 0.35 |  |  |
| PPI | 0.922(0.5297-1.605) | 0.77 |  |  |
| Statins | NA | 1 |  |  |
| **Clinical laboratory characteristics** | | | | |
| HDLC, mmol/L | 0.6019(0.1560-2.323) | 0.46 |  |  |
| LDLC, mmol/L | 0.5531(0.3663-0.8352) | 0.005** | 0.15% |  |
| Triglycerides, mmol/L | 0.953(0.6929-1.311) | 0.77 |  |  |
| HbA1c, % total haemoglobin | 0.8424(0.6184-1.147) | 0.28 |  |  |
| ALT, U/L | 1.003(0.997-1.008) | 0.36 |  |  |
| AST, U/L | 0.9909(0.9817-1.000) | 0.054 |  |  |
| CREA, umol/L | 0.9973(0.9892-1.005) | 0.52 |  |  |
| CK, U/L | 0.9969(0.9935-1.000) | 0.06 |  |  |
| CKMB, U/L | 0.9633(0.9274-1.001) | 0.054 |  |  |

*P* values were calculated by multivariate Cox regression analysis. *P* < 0.05 was considered a statistically significant difference. β represents exp(coefficient).

BMI denotes Body-mass index; MI myocardial infarction; ACEI angiotensin-converting-enzyme inhibitor; ARB angiotensin receptor blocker; BBI β-blockers inhibitors; CCB calcium channel blockers; PPI proton pump inhibitors; HDLC highdensity lipoprotein cholesterol; LDLC low-density lipoprotein cholesterol; HbA1c hemoglobin A1c; ALT alanine aminotransferase; AST aspartate aminotransferase; CREA creatinine; CK creatine kinase and CKMB creatine kinase MB.

Signif. codes: 0 '***' 0.001 '**' 0.01 '*' 0.05 '.' 0.1 ' ' 1

# Table S3. Baseline and clinical characteristics contributed to MACE in replication cohort (n=1,703).

| **Characteristics** | **β (95% CI)** | **Pvalue** | **Variants**  **explanation** |  |
| --- | --- | --- | --- | --- |
| Age – years, mean (s.d.) | 1.024(1.006-1.042) | 0.008** | 0.9% |  |
| Sex, Men – no. (%) | 1.049(0.676-1.627) | 0.83 |  |  |
| BMI –kg m–2, mean (s.d.) | 1.011(0.9442-1.084) | 0.75 |  |  |
| **Risk factors, n (%)** | | | | |
| Previous MI | 1.102(0.7655-1.587) | 0.6 |  |  |
| Diabetes mellitus | 1.153(0.7867-1.691) | 0.47 |  |  |
| Hypertension | 1.404(0.9714-2.031) | 0.07 |  |  |
| **Medications used before event** | | | | |
| ACEI_ARB | 1.261(0.7646-2.078) | 0.36 |  |  |
| BBI | 1.134(0.6498-1.978) | 0.66 |  |  |
| CCB | 1.173(0.7773-1.770) | 0.45 |  |  |
| PPI | 1.223(0.859-1.742) | 0.26 |  |  |
| Statins | 0.7647(0.2823-2.071) | 0.6 |  |  |
| **Clinical laboratory characteristics** | | | | |
| HDLC, mmol/L | 0.7509(0.3517-1.603) | 0.46 |  |  |
| LDLC, mmol/L | 0.9513(0.782-1.157) | 0.62 |  |  |
| Triglycerides, mmol/L | 0.9577(0.8016-1.144) | 0.63 |  |  |
| HbA1c, % total haemoglobin | 0.9199(0.785-1.078) | 0.3 |  |  |
| ALT, U/L | 1.002(0.9991-1.005) | 0.17 |  |  |
| AST, U/L | 0.9977(0.9928-1.003) | 0.36 |  |  |
| CREA, umol/L | 1.003(1.002-1.005) | <0.0001*** | 0.7% |  |
| CK, U/L | 0.9996(0.999-1.000) | 0.24 |  |  |
| CKMB, U/L | 0.9858(0.9656-1.006) | 0.18 |  |  |

*P* values were calculated by multivariate Cox regression analysis. *P* < 0.05 was considered a statistically significant difference. β represents exp(coefficient).

BMI denotes Body-mass index; MI myocardial infarction; ACEI angiotensin-converting-enzyme inhibitor; ARB angiotensin receptor blocker; BBI β-blockers inhibitors; CCB calcium channel blockers; PPI proton pump inhibitors; HDLC highdensity lipoprotein cholesterol; LDLC low-density lipoprotein cholesterol; HbA1c hemoglobin A1c; ALT alanine aminotransferase; AST aspartate aminotransferase; CREA creatinine; CK creatine kinase and CKMB creatine kinase MB.

Signif. codes: 0 '***' 0.001 '**' 0.01 '*' 0.05 '.' 0.1 ' ' 1

#

# Table S4. Data production summary.

| Exome sequencing | | | |  |
| --- | --- | --- | --- | --- |
|  | MACE | Without MACE | Total | |
| #of individuals | 51 | 117 | 168 | |
| Raw base (Gb) | 19.23(2.40) | 18.32(2.59) | 18.60(2.56) | |
| Mapped base (Gb) | 15.77(2.13) | 15.42(2.14) | 15.53(2.14) | |
| Mapped base on target (Gb) | 9.58(1.69) | 9.14(1.62) | 9.27(1.65) | |
| Capture specificity (%) | 61.10(10.03) | 59.78(9.92) | 60.19(9.94) | |
| Average coverage depth | 216.53(38.20) | 206.36(37.00) | 209.45(37.55) | |
| coverage>=1x (%) | 99.57(0.07) | 99.58(0.08) | 99.58(0.08) | |
| coverage>=2x (%) | 99.39(0.09) | 99.41(0.10) | 99.40(0.10) | |
| coverage>=4x (%) | 99.11(0.12) | 99.13(0.12) | 99.12(0.12) | |
| coverage>=8x (%) | 98.66(0.19) | 98.68(0.17) | 98.67(0.18) | |
| coverage>=10x (%) | 98.45(0.23) | 98.47(0.21) | 98.46(0.21) | |
| Targeted sequencing | | | |  |
|  | MACE | Without MACE | Total | |
| #of individuals | 123 | 1,580 | 1,703 | |
| Raw base (Gb) | 2.49±0.68 | 2.48±0.70 | 2.48±0.71 | |
| Mapped base (Gb) | 1.20±0.39 | 1.21±0.39 | 1.21±0.40 | |
| Mapped base on target (Gb) | 0.56±0.18 | 0.56±0.18 | 0.56±0.19 | |
| Captured specificity (%) | 47.82±7.50 | 48.89±7.99 | 48.85±7.98 | |
| Average coverage depth | 92.74±29.80 | 93.29±30.26 | 93.64±30.90 | |
| coverage>=1x (%) | 94.26(0.78) | 94.31(0.78) | 94.32(0.80) | |
| coverage>=2x (%) | 92.86(1.13) | 92.94(1.11) | 92.94(1.12) | |
| coverage>=4x (%) | 90.40(1.77) | 90.51(1.71) | 90.52(1.73) | |
| coverage>=8x (%) | 86.02(2.98) | 86.20(2.85) | 86.22(2.88) | |
| coverage>=10x (%) | 83.94(3.56) | 84.15(3.39) | 84.17(3.43) | |

1. Raw bases: number of bases in original FASTQ file, showing the yield of sequencing data.
2. Mapped base: number of bases mapped to reference genome.
3. Mapped base on target: number of reads mapped to target regions wholly or partially.
4. Captured specificity: (number of unique reads overlapping with target region)/(number of all unique reads mapped to reference genome).
5. Average coverage depth: (number of bases mapped to target region)/(size of target region).
6. coverage>=1x (%): (size of target region covered by at least 1 read)/(size of target region).
7. coverage>=2x (%): (size of target region covered by at least 2 reads)/(size of target region).
8. coverage>=4x (%): (size of target region covered by at least 4 reads)/(size of target region).
9. coverage>=8x (%): (size of target region covered by at least 8 reads)/(size of target region).
10. coverage>=10x (%): (size of target region covered by at least 10 reads)/(size of target region).

# Table S5. A total of 6MB targeted region in replication cohort.

| Method | Cut-off | Methods | SNPs | Genes |
| --- | --- | --- | --- | --- |
| Single variants association analysis | *P* < 0.05 | logistic regression analysis was performed to calculate the P values and odds ratio (OR) of SNPs on the 51 Cases with MACE and 117 Controls without MACE  (adjusting 17 covariates including principal components PC1-PC4, sex, age, BMI et al). | 6,268 |  |
| Gene-based association analysis | *P* < 0.05 | Gene-based analysis was performed using the Fast Association Tests (FAST) tool based on SNPs significance in single variants association analysis. |  | 408 |
| Known drug response related genes | Significant | Known drug response related genes involved in pharmacokinetic and pharmacodynamics pathway of clopidogrel, aspirin, statin or beta-blockers. |  | 49 |
| Total targeted region | 6MB | | | |

The targeted region consisted of three parts. The first part was the top 6,268 associated SNVs with *P* < 0.05 in the discovery cohort. The second part was top 408 genes with *P* < 0.05 in gene-based test in the discovery cohort. The third part was 49 reported genes within pharmacokinetic and pharmacodynamics pathway of clopidogrel, aspirin, statin or beta-blockers. All the three parts were merged into 6MB target regions.

# Table S6. Functional annotation of the eight significant genetic variants.

| **SNP ID** | **Located Gene** | **location** | **Proxy SNPs** | **Expression quantitative trait loci (eQTL) target gene** | **Chromatin immunoprecipitation (ChIP-seq)** | **Major adverse cardiac events affected by gene expression** |
| --- | --- | --- | --- | --- | --- | --- |
| rs17064642 | *MYOM2* | exon; synonymous | rs34823600  (r^2^ = 0.57, distance=16 bp, missense SNP) | / | / | *MYOM2* is down-regulated in cardiac hypertrophy in rats[26], in acute myocardial infarction (AMI) patients[27], and in chronic heart failure (HF) patients[28]. |
| rs11640115 | *WDR24* | intron | rs763053  (r² = 0.96,  distance=127 bp,  synonymous SNP) | LA16c-349E10.1; WDR24; FBXL16; C16orf13; WDR90; RHBDL1; | *POLR2A* | WDR90 affected MI recurrence [19]. |
| rs74569896 | *NECAB1* | intron | rs73694346  (r² = 0.92,  distance=30K bp,  3'-UTR SNP) | / | *GATA3* | Lack of *GATA3* results in conotruncal heart anomalies in mouse [25]. |
| rs4736529 | *EFR3A* | UTR3 | / | / | / | *EFR3A* was significantly upregulated in the CAD patients compared with the control group [24]. |
| rs75750968 | *AGAP3* | intron | / | / | / | / |
| rs3749187 | *ZDHHC3* | intron | / | / | / | */* |
| rs140410716 | *ECHS1* | exon; synonymous | / | / | / | *ECHS1* is down-regulated in myocardial infarction (MI) rats[20,21] and in heart failure (HF) patients (*ECHS1* protein experiments). |
| rs201441480 | *KRTAP10-4* | exon; missense | / | / | *ZNF263* | / |

The references were listed in the body text.

| **Table S7. Eight genes and their differently expression** between MI groups and sham groups among four studies. | | | | | | | | | | | | | |
| --- | --- | --- | --- | --- | --- | --- | --- | --- | --- | --- | --- | --- | --- |
| Associations | Inner or Regulated Gene | GSE7487 [1] | | | GSE47495 [2] | | | GSE48060 [3] | | | GSE27962 [4] | | |
|  |  | Sig. (2-tailed) | Mean Difference | Std. Error Difference | Sig. (2-tailed) | Mean Difference | Std. Error Difference | Sig. (2-tailed) | Mean Difference | Std. Error  Difference | Sig. (2-tailed) | Mean Difference | Std. Error Difference |
| rs17064642 | *MYOM2* | 0.316 | -3.358 | -3.445 | / | / | / | 0.968 | -0.013 | -0.260 | / | / | / |
| rs11640115 | *WDR24* | 0.091 | -1.516 | -1.375 | 0.591 | -0.040 | -0.016 | 0.082 | 0.099 | 0.063 | / | / | / |
|  | *WDR90* | 0.260 | 1.118 | 0.523 | / | / | / | **4.78E-03** | 0.122 | 0.031 | / | / | / |
|  | *WFIKKN1* | 0.489 | -0.971 | -0.888 | 0.239 | -0.078 | -0.018 | 0.052 | 0.151 | 0.058 | / | / | / |
| rs74569896 | *NECAB1* | 0.790 | 0.476 | -1.224 | 0.832 | -0.024 | 0.088 | 0.553 | -0.028 | 0.033 | / | / | / |
| rs4736529 | *EFR3A* | 0.203 | 1.997 | -1.662 | 0.281 | -0.035 | -0.015 | 0.055 | -0.199 | -0.042 | **6.94E-04** | -0.375 | 0.007 |
| rs75750968 | *AGAP3* | 0.406 | 1.142 | 0.948 | 0.745 | 0.021 | -0.007 | 0.219 | 0.049 | -0.067 | / | / | / |
| rs3749187 | *ZDHHC3* | 0.039 | 0.634 | 0.498 | 0.345 | 0.056 | -0.028 | 0.065 | 0.114 | 0.054 | / | / | / |
| rs140410716 | *ECHS1* | **5.46E-06** | -3.534 | -2.651 | **7.28E-04** | -0.237 | 0.075 | 0.408 | 0.050 | 0.004 | 0.191 | -0.306 | 0.098 |
| rs201441480 | *KRTAP10-4* | / | / | / | / | / | / | / | / | / | / | / | / |

[1] Lin RC, Weeks KL, Gao XM, et al. PI3K(p110 alpha) protects against myocardial infarction-induced heart failure: identification of PI3K-regulated miRNA and mRNA. Arterioscler Thromb Vasc Biol 2010;30:724-32.

[2] Tulacz D, Mackiewicz U, Maczewski M, Maciejak A, Gora M, Burzynska B. Transcriptional profiling of left ventricle and peripheral blood mononuclear cells in a rat model of postinfarction heart failure. BMC Med Genomics 2013;6:49.

[3] Suresh R, Li X, Chiriac A, et al. Transcriptome from circulating cells suggests dysregulated pathways associated with long-term recurrent events following first-time myocardial infarction. J Mol Cell Cardiol 2014;74:13-21.

[4] Kuster DW, Merkus D, Kremer A, et al. Left ventricular remodeling in swine after myocardial infarction: a transcriptional genomics approach. Basic Res Cardiol 2011;106:1269-81.

# **Table S8. Detailed information for four GEO datasets.**

| **GEO dataset** | **GSE7487[1]** | **GSE47495[2]** | **GSE48060 [3]** | **GSE27962[4]** |
| --- | --- | --- | --- | --- |
| Organism | Mus musculus | Rattus norvegicus | Homo sapiens | [Sus scrofa](https://www.ncbi.nlm.nih.gov/Taxonomy/Browser/wwwtax.cgi?mode=Info&id=9823) |
| Experiment type | Expression profiling by array | Expression profiling by array | Expression profiling by array | Expression profiling by array |
| Experiment condition | Ntg (nontransgenic), dnPI3K (cardiac-specific transgenic model with reduced PI3K activity) and caPI3K (transgenic mice with increased PI3K activity) mice were used | Myocardial infarction (MI) was induced in male Wistar rats by ligation of the proximal left coronary artery. The sham-operated group (control group) was subjected to the same protocol, except that the suture was not tied around the proximal left coronary artery. | blood samples from normal cardiac function controls and first-time AMI patients within 48-hours post-MI | animals that underwent either sham surgery or permanent ligation of the left coronary artery (MI). RNA was isolated from the remote, non-ischemic, regions of the left ventricle. |
| Overall design | Mice were divided into MI (myocardial infarction) group and sham (control operation) groups, resulting in 6 experimental groups. 4 arrays were included in each group, each assigned an array number as follows: | Sham-operated rats (n=6) and rats with small (n=6), moderate (n=6), and large (n=5) MI size were included into the experiment two months after the operation. Then, left ventricules and blood samples were obtained for RNA extraction and hybridization on Affymetrix microarrays. Microarrays were used to compare the LV and PBMCs transcriptomes of control and experimental animals. The development of heart failure was estimated by echocardiography and catheterization. | The overall experimental design includes blood samples from 21 control and 31 myocardial infaction patient groups. Among the 31patients, 5 patients have recurrent events. Microarray were peformed on the blood samples and comparisons of control vs patient and patients with recurrent events vs patients without recurrent events were performed to identify differential genes related to disease or patients groups with recurrent events for the following bioinformatic analysis. | RNA was isolated from 8 sham and 8 MI animals three weeks after surgery. Each group contained 4 males and 4 females. Animals used for the study were 2-3 months old Yorkshire x Landrace swine. Only neutered males entered the study. |

[1] Lin RC, Weeks KL, Gao XM, et al. PI3K(p110 alpha) protects against myocardial infarction-induced heart failure: identification of PI3K-regulated miRNA and mRNA. Arterioscler Thromb Vasc Biol 2010;30:724-32.

[2] Tulacz D, Mackiewicz U, Maczewski M, Maciejak A, Gora M, Burzynska B. Transcriptional profiling of left ventricle and peripheral blood mononuclear cells in a rat model of postinfarction heart failure. BMC Med Genomics 2013;6:49.

[3] Suresh R, Li X, Chiriac A, et al. Transcriptome from circulating cells suggests dysregulated pathways associated with long-term recurrent events following first-time myocardial infarction. J Mol Cell Cardiol 2014;74:13-21.

[4] Kuster DW, Merkus D, Kremer A, et al. Left ventricular remodeling in swine after myocardial infarction: a transcriptional genomics approach. Basic Res Cardiol 2011;106:1269-81.

# **Table S9. Statistic power for the eight top SNPs associated with MACE.**

| Gene | Chr. | Position | SNP | RAF | HR | Power |
| --- | --- | --- | --- | --- | --- | --- |
| *MYOM2* | chr8 | 2017415 | rs17064642 | 0.0644 | 2.40 | 0.972 |
| *WDR24* | chr16 | 735794 | rs11640115 | 0.6941 | 2.08 | 0.997 |
| *NECAB1* | chr8 | 91937609 | rs74569896 | 0.1538 | 1.90 | 0.972 |
| *EFR3A* | chr8 | 133023163 | rs4736529 | 0.0600 | 2.41 | 0.966 |
| *AGAP3* | chr7 | 150825806 | rs75750968 | 0.0211 | 3.21 | 0.927 |
| *ZDHHC3* | chr3 | 44986574 | rs3749187 | 0.0268 | 2.99 | 0.938 |
| *ECHS1* | chr10 | 135182452 | rs140410716 | 0.0149 | 3.32 | 0.875 |
| *KRTAP10-4* | chr21 | 45994608 | rs201441480 | 0.0111 | 3.86 | 0.881 |

R package survSNP (version 0.23.2) was used to evaluate the power for association SNPs with HR and RAF in replication cohort.

# **Table S10. Baseline characteristics of ACS patients with heart failure (HF) symptoms at NYHA stage II or less, stage III or IV.**

| **Characteristics** | **HF symptoms** | |  | **Univariable Analysis** | |
| --- | --- | --- | --- | --- | --- |
|  | **stage II or less (n=61)** | **stage III or IV (n=89)** |  | **OR (95%CI)** | ***P*** |
| Sex, Men – no. (%) | 46(75.41) | 78(87.64) |  | 2.312(0.979-5.459) | 0.0558 |
| Age – yrs, mean (±s.d.) | 64.86±10.79 | 66.95±9.13 |  | 1.022(0.988-1.057) | 0.202 |
| **Risk factors, n (%)** | |  |  |  |  |
| Diabetes mellitus | 14(22.95) | 28(31.46) |  | 1.541(0.731-3.249) | 0.2558 |
| Hypertension | 32(52.46) | 60(67.42) |  | 1.875(0.959-3.664) | 0.066 |
| **Medications used before event, n (%)** | | | | |  |
| ACEI | 25(40.98) | 62(69.66) |  | 3.307(1.673-6.538) | 0.0006 |
| BB | 52(85.25) | 75(84.27) |  | 0.928(0.374-2.302) | 0.8712 |
| CCB | 17(27.87) | 45(50.56) |  | 2.647(1.318-5.315) | 0.0062 |
| PPI | 31(50.82) | 65(73.03) |  | 2.621(1.319-5.206) | 0.0059 |
| Statins | 60(98.36) | 85(95.51) |  | 0.354(0.039-3.248) | 0.3586 |
| **Clinical laboratory characteristics, mean (s.d.)** | | | | |  |
| HDLC, mmol/L | 1.04±0.29 | 0.92±0.23 |  | 0.155(0.036-0.662) | 0.0119 |
| LDLC, mmol/L | 2.68±0.73 | 2.65±0.95 |  | 0.961(0.651-1.42) | 0.8421 |
| Triglycerides, mmol/L | 1.82±0.95 | 1.37±0.81 |  | 0.549(0.36-0.839) | 0.0056 |
| CHOL, mmol/L | 4.52±0.86 | 4.2±1.16 |  | 0.746(0.538-1.032) | 0.0768 |
| ALT, U/L | 29.35±16.92 | 35.42±33.13 |  | 1.009(0.994-1.024) | 0.2271 |
| AST, U/L | 34.24±31.57 | 39.89±37.3 |  | 1.005(0.995-1.016) | 0.3479 |
| CREA, umol/L | 84.83±20.9 | 137.75±97.11 |  | 1.034(1.018-1.051) | <.0001 |
| CK, U/L | 181.74±378.46 | 171.86±256.45 |  | 1(0.999-1.001) | 0.8532 |
| CKMB, U/L | 8.66±13.42 | 9.25±8.01 |  | 1.005(0.973-1.039) | 0.7459 |
| **Gene** |  |  |  |  |  |
| *ECHS1* | 215.18±115.67 | 161.84±76.67 |  | 0.419(0.248-0.709) | 0.0012 |

P values were calculated by logistic regression analysis. P < 0.05 was considered a statistically significant difference.

MI myocardial infarction; ACEI angiotensin-converting-enzyme inhibitor; ARB angiotensin receptor blocker; BBI β-blockers inhibitors; CCB calcium channel blockers; PPI proton pump inhibitors; HDLC high density lipoprotein cholesterol; LDLC low density lipoprotein cholesterol; HbA1c hemoglobin A1c; ALT alanine aminotransferase; AST aspartate aminotransferase; CREA creatinine; CK creatine kinase and CKMB creatine kinase MB. *ECHS1* Enoyl-CoA Hydratase, Short Chain 1.

# Table S12. Association results of five previously reported SNVs associated with clopidogrel response.

| Variants | SNP | Effect allele | Not effect allele | Discovery | | Replication | | Meta *P* |
| --- | --- | --- | --- | --- | --- | --- | --- | --- |
|  |  |  |  | HR | *P* | HR | *P* |  |
| *CYP2C19*2* (681G>A) | rs4244285 | A | G | 1.80 | 0.044 | 0.87 | 0.345 | 0.766 |
| *CYP2C19*3* (636G>A) | rs4986893 | A | G | 0.34 | 0.176 | 0.86 | 0.619 | 0.379 |
| *CYP2C9*3* (1075A>C) | rs1057910 | C | A | 1.03 | 0.958 | 0.87 | 0.686 | 0.712 |
| *ABCB1* (3435C>T) | rs1045642 | T | C | 0.92 | 0.762 | 1.14 | 0.321 | 0.392 |
| *PON1*(Q192R,576A>G) | rs662 | G | A | 1.27 | 0.378 | 1.49 | 0.006 | 0.0036 |


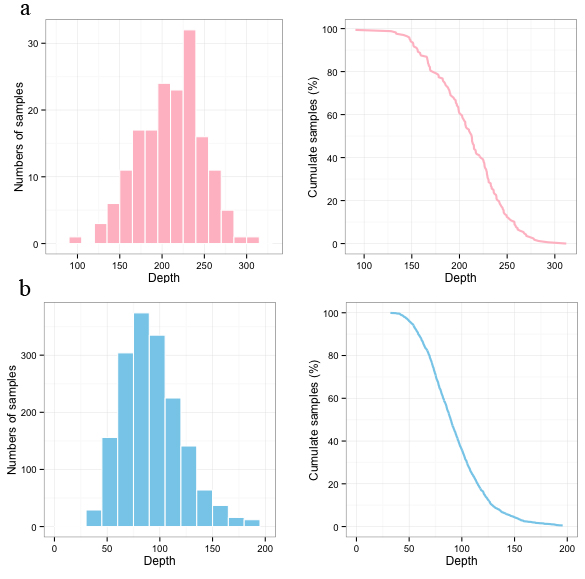


# Figure S1. Depth distribution of sequencing.

Figure on left is the distribution of sequencing depth of all samples in targeted region. X-axis denotes sequencing depth, while Y-axis indicated the number of samples under a given range of sequencing depth. Figure on right is cumulative depth distribution. X-axis denotes sequencing depth, and Y-axis indicated the fraction of samples that achieves at or above a given sequencing depth. (a) Depth of exome sequencing for 168 individuals in discovery cohort. (b) Depth of targeted sequencing for 1,703 individuals in replication cohort.


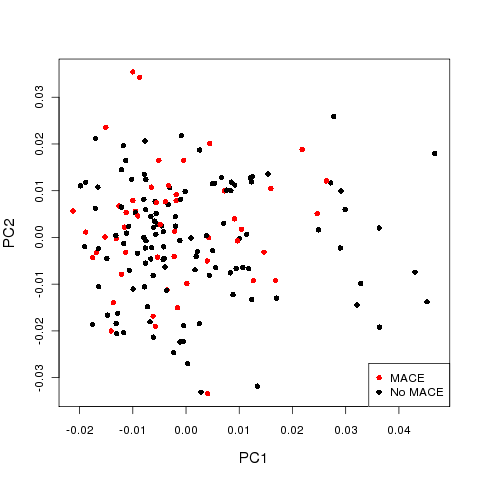


a


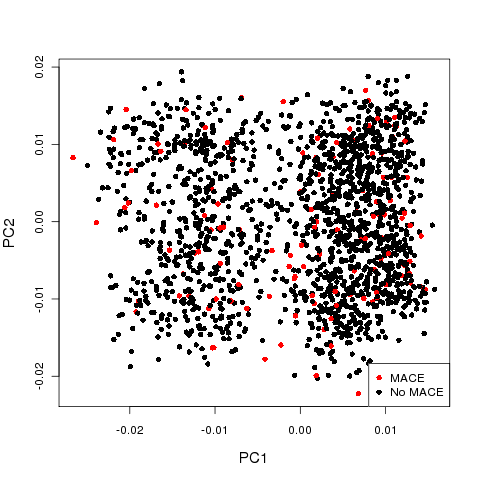


b

# Figure S2. PCA analysis in discovery and replication cohort, respectively.

(a) PCA analysis for 51 cases with MACE and 117 controls without MACE in discovery cohort with exome sequencing. (b) PCA analysis for 123 cases with MACE and 1,580 controls without MACE in replication cohort with targeted sequencing.


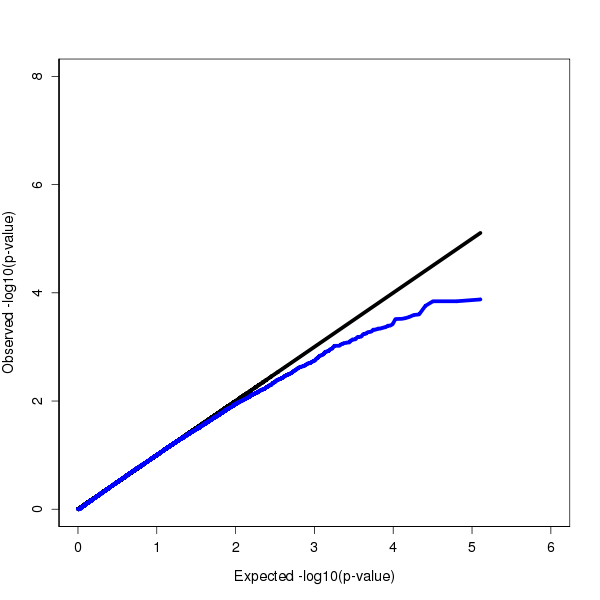


**a**


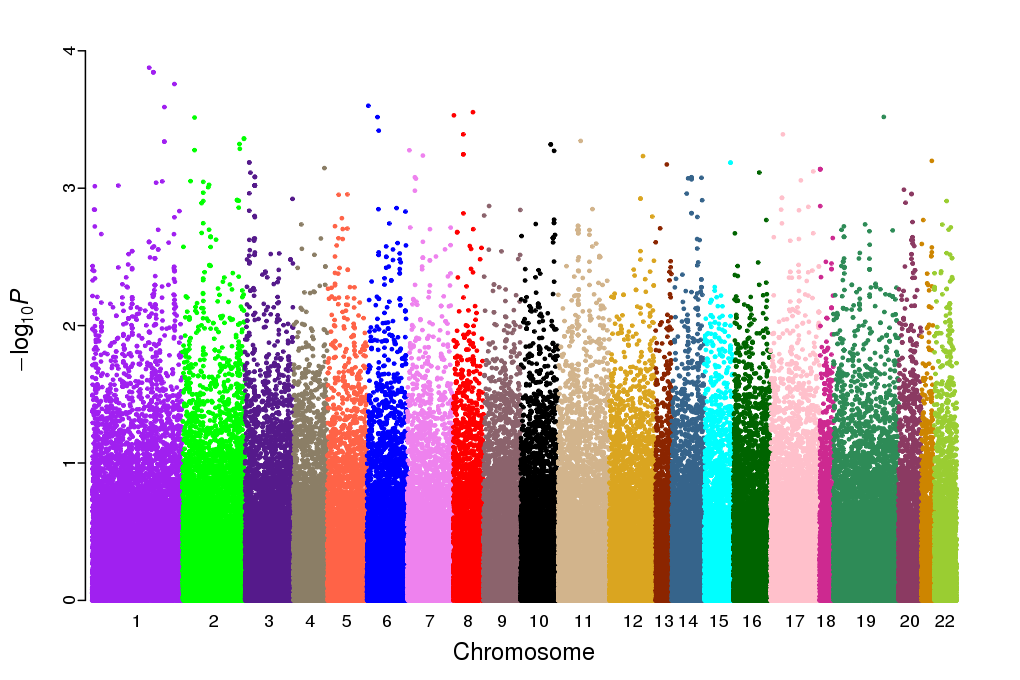


**b**


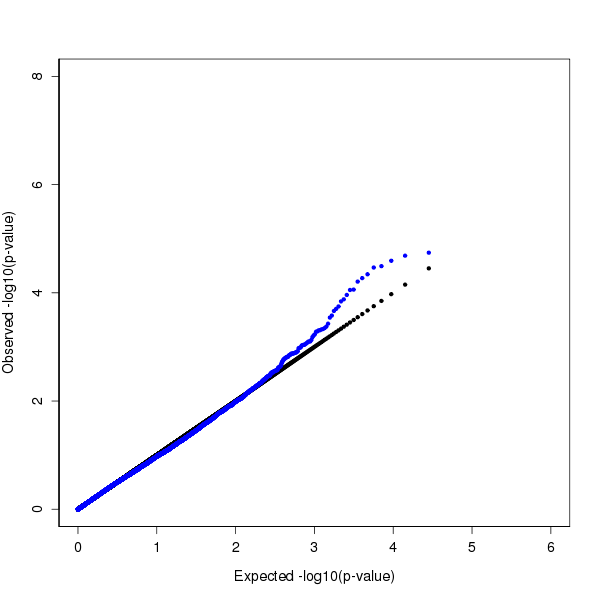


**c**


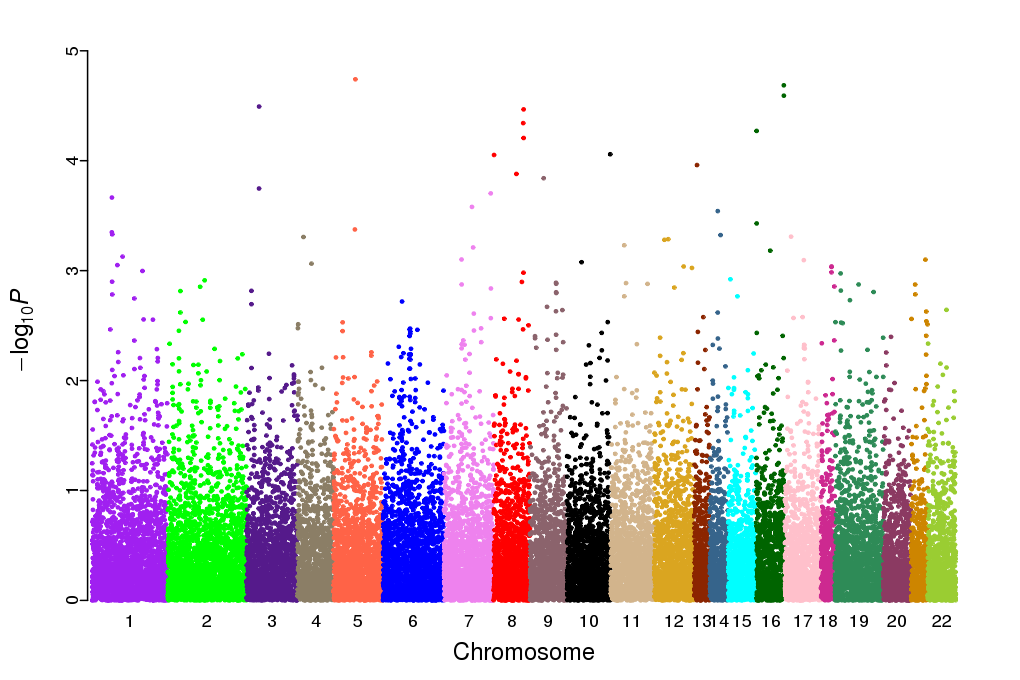


**d**

# Figure S3. QQ plot and Manhattan plot of associations for MACE.

LD Score regression analysis was perform using ldsc tool (doi: 10.1038/ng.3211).

(a-b) QQ plot (lambda =0.991; LDSC Intercept (SE)=1.001(0.012)) and Manhattan plot of SNVs in discovery cohort (exome sequencing).

(c-d) QQ plot (lambda =1.07; LDSC Intercept (SE)=1.023(0.018)) and Manhattan plot of SNVs in replication cohort (targeted sequencing).


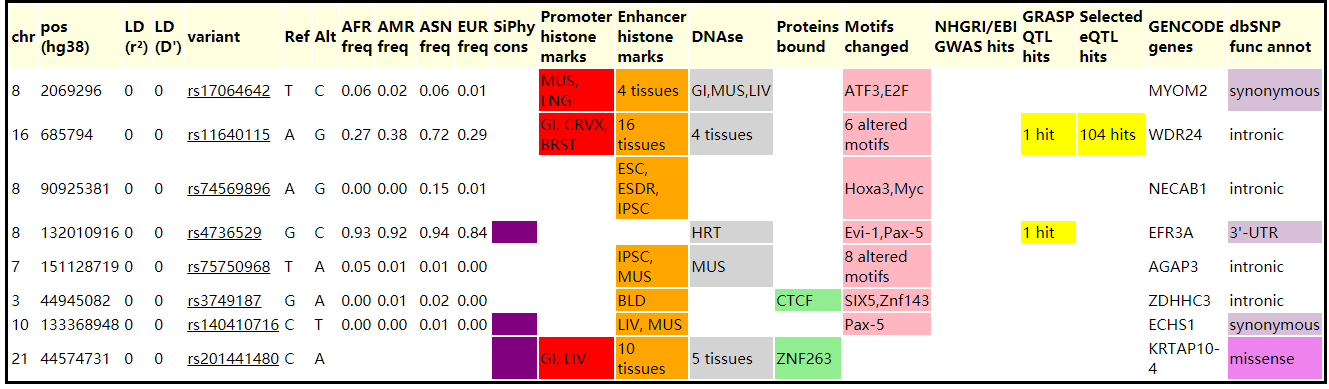


# Figure S4. HaploReg results for the eight significant SNPs associated with MACE.

The annotations of these SNPs were searched from haploreg website (https://pubs.broadinstitute.org/mammals/haploreg/haploreg.php).


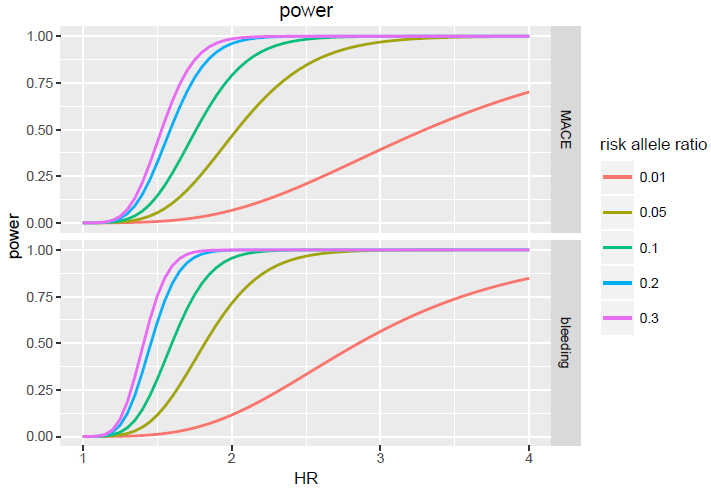


# Figure S5. Statistics power.

Power analysis was used to investigate if we have enough power to detect the associated SNPs in the sample size of replication cohort. R package survSNP (version 0.23.2) was used to evaluate the power for SNPs with different hazard ratio (HR) or risk allele ratio.


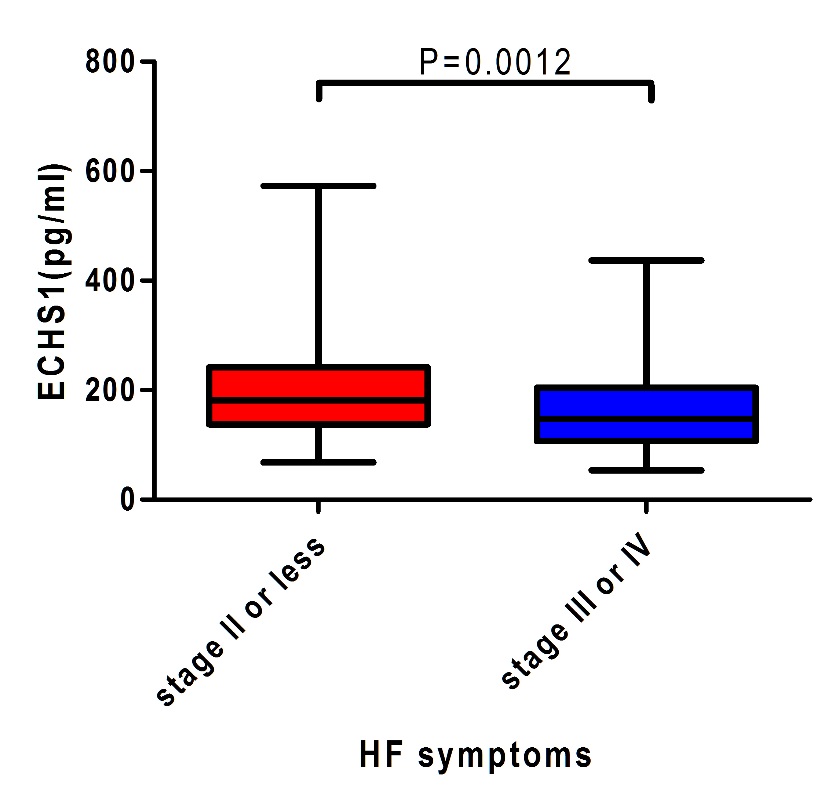


# Figure S6. Compared plasma ECHS1 level in ACS patients with severe HF symptoms (stage III or IV) to those with less serious HF symptoms (stage II or less).

We randomly selected ACS patients with HF symptoms at NYHA stage II or less (n=61), stage III or IV (n=89) from an independent study cohort. All ACS patients were sequentially enrolled in Guangdong General Hospital between June 2012 and Apr 2014 according to the same inclusion and exclusion criteria as in the discovery stage and follow-up stage. The diagnosis of HF was established according to the diagnostic criteria for HF proposed by the NYHA.

Blood samples were collected and anticoagulated with ethylenediamine tetraacetic acid (EDTA) dipotassium salt. The plasma was isolated within an hour by centrifugation at 3000 rpm for 10 min at 4°C to retrieve plasma, and then stored at −80°C until assayed. *ECHS1* protein levels was measured in plasma sample using sandwich enzyme-linked immunosorbent assays (ELISA) (ECHS1 ELISA kit, Action-award Biotech co. Ltd., Guangzhou, China) and a Multiskan GO Microplate Reader (Thermo Scientific Inc., USA); 10pg/ml -700pg/ml was considered within the normal range.

The comparison of parameters and *ECHS1* protein levels between patients with HF at stage II or less and patients at stage III or IV was performed using the logistic regression analysis. Because the distribution of *ECHS1* protein levels was skewed, logarithmic transformation was performed before analysis.


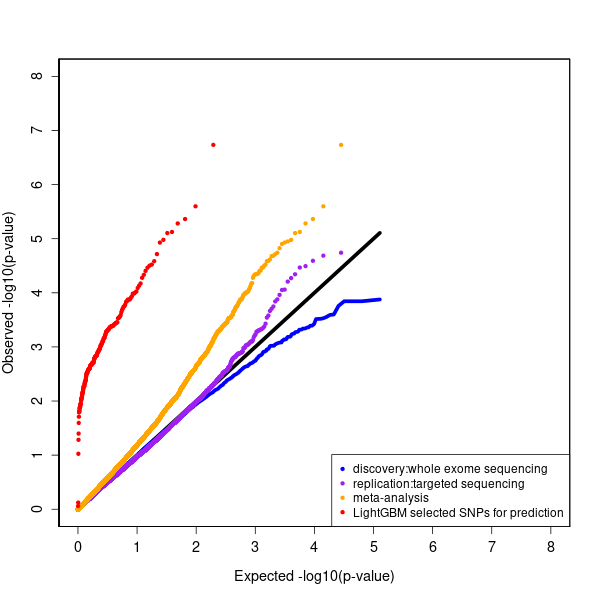


# Figure S7. The QQ plots for the discovery SNPs, replication SNPs, the meta-analysis SNPs and the SNPs selected by LightGBM for prediction of 18-months MACE in this study.

# Those SNPs effectively contributed to the prediction have significant small P-value enrichment in our GWAS result.
